# How to treat primary cutaneous B cell lymphoma – Results from a monocentric cohort study on 98 patients

**DOI:** 10.1111/ddg.15702

**Published:** 2025-05-30

**Authors:** Rohat Cankaya, Pit Leonard Kleiner, Franz Joachim Hilke, Rose Moritz, Thomas Eigentler, Max Schlaak, Gabor Dobos

**Affiliations:** ^1^ Department of Dermatology Venereology and Allergology Charité – Universitätsmedizin Berlin Berlin Germany; ^2^ Skin Cancer Centre Charité – Universitätsmedizin Berlin Berlin Germany

**Keywords:** CBCL, cutaneous B cell lymphoma, cutaneous T cell lymphoma, excision, patient survival, prognostic factors, Primary cutaneous lymphoma, radiotherapy, systemic treatments, time to next treatment (TTNT), triamcinolone

## Abstract

**Background:**

Primary cutaneous B cell lymphomas (CBCL) are chronic diseases with frequent relapses. Time to next treatment (TTNT) is an endpoint reflecting clinical benefit of treatments including patient perspectives. The objectives were to evaluate clinical characteristics, survival, prognosis and TTNT in CBCL.

**Patients and methods:**

In this monocentric study, clinical data were extracted between 1998 and 2022. TTNT were calculated. Univariate and multivariate analyses were conducted.

**Results:**

Altogether, 46 patients with follicle center lymphoma (pcFCL), 41 with marginal zone lymphoproliferative disorder (pcMZLPD) and 11 with diffuse large B‐cell lymphoma, leg type (DLBCL‐LT) were identified. 26% of pcFCL patients relapsed frequently. The 5‐year relapse‐free survival was 71%, 87% and 23% in pcFCL, pcMZLPD and DLBCL‐LT, respectively. In pcFCL and pcMZLPD, skin‐directed treatments, such as excision or intralesional triamcinolone, performed best based on TTNT, while chemotherapy achieved a mean TTNT of 38 months in DLBCL‐LT. In multivariate analysis of all patients, leg involvement was significantly associated with a decreased TTNT of the first treatment, while comorbidities were associated with an increased TTNT.

**Conclusions:**

DLBCL‐LT had the worst survival. Skin‐directed treatments tend to achieve higher TTNT in pcFCL and pcMZLPD, while systemic treatments had higher TTNT in DLBCL‐LT.

## INTRODUCTION

Primary cutaneous lymphomas are a heterogeneous disease group of rare non‐Hodgkin lymphomas from either T or B lymphocytes.^1^, ^2^ The latter disease group, primary cutaneous B cell lymphomas (CBCL),^1^, ^2^ consists of the following diagnoses: Primary cutaneous follicle center lymphoma (pcFCL), primary cutaneous marginal zonal lymphoproliferative disorder (pcMZLPD) (also called primary cutaneous marginal zone lymphoma [pcMZL]),^1^ diffuse large B cell lymphoma, leg type (DLBCL‐LT) and further rare entities.^1^, ^3^, ^4^

The concept of primary cutaneous marginal zone lymphoma (pcMZL) has been adjusted several times in the past decades. Willemze et al. defined the entity as part of the extranodal marginal zone B‐cell lymphoma group, as an indolent lymphoma composed of small B cells, including marginal zone (centrocyte‐like) cells, lymphoplasmacytoid cells and plasmacells in 2005.^2^ In the current version of the WHO classification of lymphoid neoplasms, Alaggio et al. regrouped pcMZL as a separate entity distant from other marginal zone lymphomas, owing to its distinctive clinicopathological features.^3^ The international Consensus Committee went even further and downgraded pcMZL from lymphoma to a lymphoproliferative disorder (pcMZLPD) due to its indolent behavior, as disease‐specific survivals approach 100% without requiring aggressive therapies.^4^ Hence, we will refer to the disease entity as pcMZLPD.^4^

Besides different nomenclatures, there is also no consensus regarding the treatment of pcMZLPD. Apart from watchful waiting, treatment of pcMZLPD patients may be deemed necessary due to possible impairment of quality of life from visible skin lesions or itching, which can increase patients’ urge to seek treatment. In the current study, watchful waiting was done in exceptional cases only, because to our knowledge, there is no evidence supporting watchful waiting in pcMZLPD.^5^, ^6^

While pcFCL and pcMZLPD as indolent CBCL diseases entities show a good prognosis, patients with DLBCL‐LT still have a poor survival.^7^, ^8^, ^9^, ^10^, ^11^ CBCL are chronic diseases which are characterized by frequent relapses during their disease course.^7^, ^8^, ^9^, ^10^, ^11^ Evidence about overall and relapse‐free survival (OS, RFS) and prognostic factors are sparse due to paucity of clinical studies sample sizes.

According to current recommendations, skin‐directed and systemic treatments are available in treating CBCL, ranging from excision, intralesional corticosteroids to polychemotherapy.^5^, ^6^

In the last decades, rituximab and polychemotherapy as treatment regimens have improved patient survival in DLBCL‐LT.^12^ In contrast to primary cutaneous T cell lymphomas (CTCL), re‐challenge of treatment options, already given to CBCL patients, has been shown to be effective in clinical studies.^13^, ^14^

As patients frequently relapse, multiple treatment lines are often administered. Evidence about treatment efficacy is sparse due to the scarcity of clinical studies.

### Time to next treatment

Campbell et al. introduced the endpoint time to next treatment (TTNT) in clinical studies about CTCL and chronic diseases. It is measured from the start of a therapy to the start of a subsequent treatment line.^15^ TTNT is a feasible surrogate marker assessing clinical benefit, patient compliance and toxicity of treatment options reliably. In 2022, TTNT has not been evaluated in studies about CBCL.

### Prognostic factors

Both clinical and immunohistochemical biomarkers have been described as prognostic factors in CBCL following univariate and multivariate analyses, most of which included leg involvement^9^, ^16^, ^17^, ^18^ and multiple lesions.^11^, ^16^, ^18^, ^19^, ^20^

Mian et al. reported on the *Cutaneous Lymphoma International Prognostic Index* (CLIPI).^19^ In this study, they calculated a score which is based on the following parameters: Elevation in serum lactate dehydrogenase (LDH) levels, presence of nodules as primary skin lesion and a total number of lesions greater two. One point was given for each parameter if answered positively.^19^

### Objectives

The objectives of this study were to assess patient characteristics and survival, TTNT and to identify prognostic factors based on clinical parameters in CBCL patients.

## PATIENTS AND METHODS

### Study design

In this monocentric and explorative cohort study, we included eligible patients with CBCL older than 18 years treated at Charité‐Universitätsmedizin Berlin between 1998 and 2022.

After clinicopathological correlation, patients were diagnosed according to the WHO‐EORTC classifications from 2005 and 2018.^1^, ^2^

This study was conducted in accordance with the principles of the current version of the Helsinki declaration and approved by the institutional review board (IRB approval status: Reviewed and approved by local IRB Ethikkommission Berlin; approval no.: EA1/196/08).

### Data collection

Clinical data were prospectively obtained by attending physicians^21^ from medical records and included patient characteristics (age, sex and comorbidities), skin involvement at diagnosis [type, site and extent], T classification according to Kim et al.^5^) and elevated LDH levels.

Further information about the disease course, including relapses, disease progression and survival, was collected. Additionally, data regarding the treatment course with all administered treatment lines were extracted.

The *Eastern Cooperative Oncology Group performance status* (ECOG) was retrospectively assigned to each patient based on medical records.^22^

According to Mian et al. we calculated each patient a CLIPI.^19^ Therefore, two adjustments were necessary: If LDH was not assessed, a value of 0 was assigned. Instead of considering patients with more than two lesions according to Mian et al., we assigned one point to patients with more than one lesion.^19^ These adjustments were deemed necessary as LDH levels were missing in 51 CBCL patients. Additionally, we were not able to assess the number of lesions of patients since this parameter was occasionally not documented.

The data collection was retrospectively conducted, and follow‐up data was collected until June 30, 2022. Patients diagnosed after June 30, 2022, were not included.

### Time to next treatment

Standardized definitions of TTNT from Campbell et al. were respected in our study.^15^ Following adjustments were deemed necessary to calculate the TTNT in our cohort: Two identical treatment lines were considered as separate lines if the interval between them exceeded 12 months. If the onset of a treatment line is unknown, the date of discontinuation of the prior treatment line was used to determine the TTNT.

### Statistical analysis

Statistical analyses were performed in Microsoft – Excel for Mac (Version 16.74) and in IBM SPSS Statistics (Version 29).

The overall survival (OS) time was defined as time between the date of diagnosis and the date of death or last follow‐up visit. DLBCL‐LT patients were considered deceased if documented or if seen lastly in our center more than 5 years ago.

Differences between subgroups were assessed by univariate analysis with the method of Kaplan and Meier and the log rank test. Following parameters were assessed: sex, age (≥ 70 years, < 70 years), clinicopathological diagnosis, CLIPI score, elevated LDH levels, tumor nodules, site of skin lesions and T classification at diagnosis. Endpoints were OS and relapse free survival (RFS).

The multiple regression analysis was performed with the dependent variables TTNT of the first line treatment and number of relapses following statistical recommendation by Field.^23^ Variables which showed differences with a p‐value < 0.2 were included into the multivariate analysis with listwise deletion of missing data. Further variables (see data collection) were systematically included. After identification of a significant model, a stepwise removal of variables was conducted to confirm the model's robustness. Ultimately, multicollinearity and heteroskedasticity were checked.

All (two‐tailed) tests were regarded statistically significant with a p‐value < 0.05.

## RESULTS

### Patient characteristics

In total, 98 CBCL patients were identified. Their clinical characteristics are depicted in Table 1.

**TABLE 1:** Clinical characteristics of 98 patients with primary cutaneous B cell lymphoma.

Characteristics	Patients							
	*All patients*		*pcFCL*		*pcMZLPD*		*DLBCL‐LT*	
	*Absolute/total*	*%*	*Absolute/total*	*%*	*Absolute/total*	*%*	*Absolute/total*	*%*
**Total number**	98		46		41		11	
**Sex, male to female ratio**	54/44		27/19		21/20		6/5	
**Age mean (SD) [range], years**	56 (16) [20–88]		53 (12) [21–77]		48 (17) [20–80]		76 (7) [65–88]	
**ECOG performance status**								
0	57/61**^i^**	93	33/33**^j^**	100	20/22**^k^**	91	4/6**^l^**	67
1	1/61	2	0/33	0	1/22	5	0/6	0
2	1/61	2	0/33	0	0/22	0	1/6	17
3	1/61	2	0/33	0	1/22	5	0/6	0
4	0/61	0	0/33	0	0/22	0	0/6	0
5	1/61	2	0/33	0	0/22	0	1/6	17
**Comorbidities**	74/98	76	36/46	78	30/41	73	8/11	73
Immunological	28/98	29	13/46	28	15/41	37	0/11	0
Infectious	12/98	12	5/46	11	5/41	12	2/11	18
Secondary malignancies	18/98	18	10/46	22	6/41	15	2/11	18
Others	63/98	64	30/46	65	25/41	61	8/11	73
**Extent of skin involvement at initial diagnosis**								
One body region	69/98	70	39/46	85	22/41	54	8/11	73
Two body regions	9/98	9	3/46	7	5/41	12	1/11	9
More than two body regions	13/98	13	3/46	7	9/41	22	1/11	9
**T classification at initial diagnosis** ^a^								
1	36/98	37	19/46	41	12/41	29	5/11	45
2	29/98	30	15/46	33	10/41	24	4/11	36
3	16/98	16	3/46	7	12/41	29	1/11	9
**Type of skin lesions**								
Macula	3/98	3	1/46	2	2/41	5	0/11	0
Papule	4/98	4	2/46	4	2/41	5	0/11	0
Patch	16/98	16	8/46	17	6/41	15	2/11	18
Nodule	53/98	54	21/46	46	24/41	59	8/11	73
**Elevation of LDH at diagnosis**	14/51	27	5/28	18	1/16	6	3/11	27
**CLIPI**								
0	31/98	32	18/46	39	11/41	27	2/11	18
1	45/98	46	20/46	43	21/41	51	4/11	36
2	21/98	21	8/46	17	9/41	22	4/11	36
3	1/98	1	0/46	0	0/41	0	1/11	9
**First line therapy**								
Excision	34/83	41	14/40	35	14/34	41	6/9	67
Radiotherapy	14/83	17	11/40	28	3/34	9	0/9	0
Triamcinolone (intralesional)	7/83	8	4/40	10	3/34	9	0/9	0
Topical steroids	12/83	14	6/40	15	6/34	18	0/9	0
Interferon‐α	5/83	6	2/40	5	3/34	9	0/9	0
R‐CHOP	3/83	4	0/40	0	1/34	3	2/9	22
CHOP	1/83	1	0/40	0	0/34	0	1/9	11
Rituximab	4/83	5	3/40	8	1/34	3	0/9	0
**Relapse rate**	43/98	44	20/46	43	18/41	44	5/11	45
**Frequent relapser** ^b^	21/98	21	12/46	26	7/41	17	2/11	18
**5‐year relapse‐free survival,%**	72		71		87		23^c^	
**5‐year overall survival,%**	92		100		100		55	

*Abbr*.: pcFCL, primary cutaneous follicle center lymphoma; pcMZLPD, primary cutaneous marginal zone lymphoproliferative disorder; DLBCL‐LT, diffuse large B‐cell lymphoma, leg type; SD, standard deviation; ECOG, Eastern Cooperative Oncology Group; LDH, Lactate dehydrogenase; CLIPI, Cutaneous Lymphoma International Prognostic Index; R‐CHOP, Rituximab, Cyclophosphamide, Doxorubicin Hydrochloride, Vincristine Sulfate, Prednisone;

CHOP, see prior abbreviation.

^a^ Patients were staged (T classification) according to Kim et al. TNM classification system for primary cutaneous lymphomas other than mycosis fungoides and Sezary syndrome: a proposal of the International Society for Cutaneous Lymphomas (ISCL) and the Cutaneous Lymphoma Task Force of the European Organization of Research and Treatment of Cancer (EORTC). Blood. 2007.

^b^ Patients who have relapsed more than once.

^c^ Here: 5 years progress free survival.

In each subgroup, more males were identified than females. The mean ages at diagnosis were 53 years in pcFCL, 48 years in pcMZLPD and 76 years in DLBCL‐LT. Comorbid diseases and secondary malignancies were observed in 74 of 98 and 18 of 98 CBCL patients.

The ECOG score could be retrospectively determined in 61 patients, 57 of whom had a score of 0. Among patients with pcMZLPD, two patients had scores of 1 and 3, while 2 DLBCL‐LT patients had scores of 2 and 5.

Disease involvement in a single‐body region was observed in 39 of 46, 22 of 41 and 8 of 11 patients with pcFCL, pcMZLPD and DLBCL‐LT, respectively.

At diagnosis, 19 of 46 pcFCL patients, 12 of 41 pcMZLPD patients and 5 of 11 DLBCL‐LT patients were staged as T1 stage.

Initial tumor nodules were identified in 21 of 46, 24 of 41 and 8 of 11 patients with pcFCL, pcMZLPD and DLBCL‐LT, respectively. Four of 11 DLBCL‐LT patients presented with tumor ulceration.

An elevation in LDH was recorded in 5 of 28 patients with pcFCL and 1 of 16 patients with pcMZLPD. It is crucial to mention that LDH levels were only assessed in 28 pcFCL and 16 pcMZLPD patients. Furthermore, LDH levels were evaluated in 3 patients with DLBCL‐LT, all of whom had increased values.

Relapses rates were 20 of 46 pcFCL patients, 18 of 41 pcMZLPD patients and 5 of DLBCL‐LT patients, with multiple relapses occurring in 12 of 46, 7 of 41 and 2 of 11 patients in each corresponding subgroup.

The mean follow‐up time was 69 (0–282) (standard deviation: 66) months.

In one patient with DLBCL‐LT, pulmonary manifestation was found 31 months after diagnosis. Therefore, the patient was classified as M1.^5^ The patient deceased 6 months after disease progression. Another patient with DLBCL‐LT presented with nodal manifestation affecting axillary lymph nodes 11 months after diagnosis (N1 classification^5^). This patient's survival status is unknown.

### Patient survival

The 5‐year RFS was 71%, 87% and 23% in pcFCL, pcMZLPD and DLBCL‐LT, respectively, while the 5‐year OS was 100% in both pcFCL and pcMZLPD and 55% in DLBCL‐LT.

### Time to next treatment

Table 2 shows the TTNT of treatment options which were administered to the patients.

**TABLE 2:** Time to next treatment of treatment options in primary cutaneous B cell lymphoma.

Treatment options in CBCL			Mean TTNT (SD) [months]		
	*n [patients]*	*n [treatment lines]*	*pcFCL*	*pcMZLPD*	*DLBCL‐LT*
Excision	39	45	37 (37)	54 (45)	30 (15)
Radiotherapy	30	34	30 (43)	66 (86)	13 (10)
Triamcinolone (intralesional)	20	22	29 (25)	24 (46)	–
Topical steroids	31	34	19 (16)	20 (25)	–
Interferon‐α	14	16	15 (20)	18 (11)	
R‐CHOP	5	5	–	–	12 (13)
CHOP	2	2	–	–	38 (52)
Rituximab	14	16	50 (40)	7 (2)	23 (37)

*Abbr*.: n [patients], number of patients receiving the according treatment option at least once; n [treatment lines], number of treatment lines of the according treatment option; CBCL, primary cutaneous B cell lymphoma; pcFCL, primary cutaneous follicle center lymphoma; pcMZLPD, primary cutaneous marginal zone lymphoproliferative disorder; DLBCL‐LT, diffuse large B cell lymphoma, leg type; R‐CHOP, rituximab, cyclophosphamide, doxorubicin hydrochloride, vincristine sulfate, prednisone; CHOP, see prior abbreviation; TTNT, Time to next treatment; SD, standard deviation; –, not applied or not possible to determine due to sample size of one

The following treatment options were regarded skin‐directed therapies (SDT): Excision, radiotherapy, intralesional triamcinolone (TRIL) and topical steroids, while interferon (IFN)‐α, rituximab and chemotherapy (cyclophosphamide, doxorubicin hydrochloride, vincristine sulfate, prednisone [CHOP] and CHOP with rituximab [R‐CHOP]) were commonly administered systemic treatments. The dose of radiotherapy varied between 20–36 Gy.

In pcFCL, the treatments with the highest mean TTNT were rituximab (50 months, range: 9–548), excision (37 months, range: 4–526) and radiotherapy (30 months, range: 3–588). TRIL had a mean TTNT of 29 (8–308) months.

In the subgroup of pcMZLPD, radiotherapy, excision and TRIL yielded the highest mean TTNT with 66 (4–880), 54 (0.4–461) and 24 (4–649) months, respectively. In DLBCL‐LT patients, CHOP, excision and rituximab achieved the longest mean TTNT with 38 (4–326), 30 (8–211) and 23 (1–286) months, respectively (Table 2).

### Univariate analysis

**FIGURE 1:**
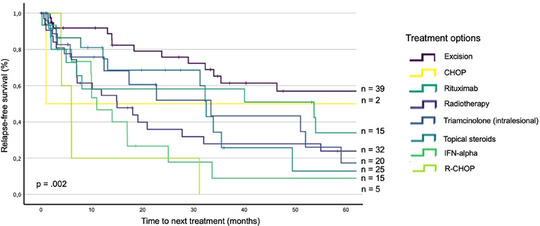
Kaplan Meier curve about relapse‐free survival of treatment options in primary cutaneous B cell lymphoma (univariate analysis). Relapse‐free survival of all treatment lines applied to patients with primary cutaneous B cell lymphoma were analyzed. *Abbr*.: CHOP, Cyclophosphamide, Doxorubicin Hydrochloride, Vincristine Sulfate, Prednisone. IFN‐alpha, Interferon‐alpha. R‐CHOP, Rituximab, Cyclophosphamide, Doxorubicin Hydrochloride, Vincristine Sulfate, Prednisone. p, p‐value (Level of significance: α = 0.05). n, number of treatment lines.

In a univariate analysis with the method of Kaplan Meier, the 5‐year RFS were calculated, which were as follows for each administered treatment option: Excision (58%), CHOP (50%), rituximab (34%), radiotherapy (24%), TRIL (18%), topical steroids (15%), IFN‐α (10%) and R‐CHOP (0%). (*p*  =  0.002) (Figure 1).

**FIGURE 2:**
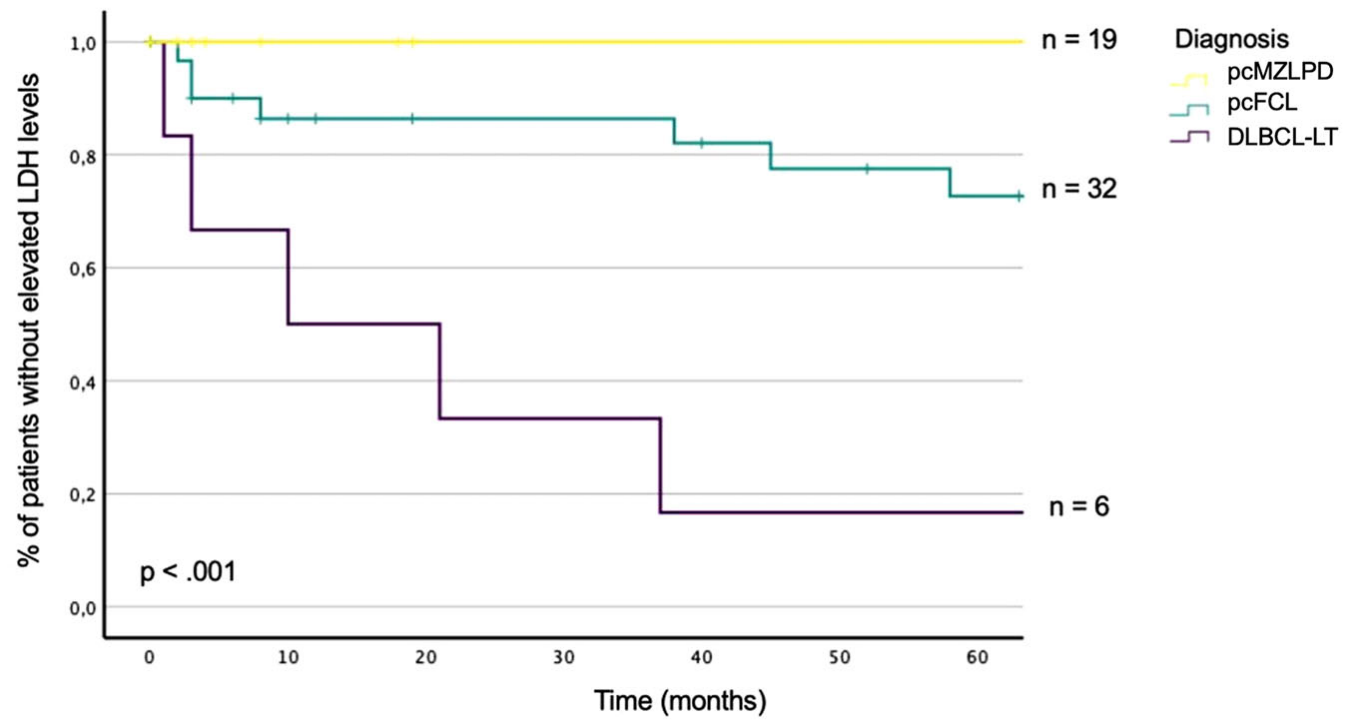
Kaplan Meier curve about elevated lactate dehydrogenase levels in primary cutaneous B cell lymphoma (univariate analysis). Differences of elevated lactate dehydrogenase levels are visualized between clinicopathological diagnoses of primary cutaneous B cell lymphoma. *Abbr*.: LDH, Lactate dehydrogenase. MZLPD, marginal zone lymphoproliferative disorder. FCL, Follicle center lymphoma. DLBCL, LT, diffuse large B‐cell lymphoma, leg type. p, p‐value (Level of significance: α = 0.05). n, number of patients.

In another univariate analysis, 26% of patients with pcFCL and 83% of patients with DLBCL‐LT showed an elevation in LDH levels. All patients with pcMZLPD had LDH levels within normal limits (*p* < 0.001) (Figure 2).

Further univariate analyses revealed significant differences in arm and leg involvement among the clinicopathological diagnoses within CBCL (online supplementary Figures S1, S2).

No significant differences in OS and RFS were observed between subgroups stratified by sex, age, elevated LDH levels, CLIPI score, tumor nodules, T classification, and lesion site.

### Prognostic factors

In multivariate analysis, leg involvement of all patients at initial diagnosis, pcFCL as diagnosis and comorbidities had a statistically significant impact on the TTNT of the first line treatment.

Topical steroids as first line treatment, trunk and lower body involvement (body regions below the umbilicus, defined by Kim et al.^5^) at diagnosis and secondary malignancies were statistically significant of the dependent variable number of relapses (Table 3).

**TABLE 3:** Multivariate analyses of time to next treatment and number of relapses in primary cutaneous B cell lymphoma.

Parameter	Multivariate analysis			
	*R^2^*	*Coefficient*	*B*	*p*
**Time to next treatment, first treatment**				
Leg involvement at diagnosis	19.2%	−125.737	−0.287	0.013
Primary cutaneous follicle center lymphoma		−90.885	−0.284	0.013
Comorbidities, immunological		78.593	0.220	0.043
Comorbidities, non‐infectious or immunological		96.351	0.280	0.010
**Number of relapses**				
Topical steroids as first line treatment	35.0%	−2.362	−0.406	0.005
Lower body involvement at diagnosis		−1.818	−0.451	0.004
Secondary malignancies		−1.277	−0.317	0.022
Relapse‐free survival time		0.009	0.178	0.184
Trunk involvement at diagnosis		1.064	0.301	0.036

*Abbr*.: R^2^, Adjusted R‐squared; coefficient, regression coefficient B; B, standardized coefficient B; p‐value (Level of significance: α = 0.05)

## DISCUSSION

In this cohort of CBCL, we report on recent data on patient characteristics, prognostic factors and treatment outcome of diverse treatment options applying the clinical endpoint TTNT.

Our patient characteristics are comparable to cohorts described previously in the literature, particularly concerning age, sex and extent of cutaneous involvement.^8^, ^11^, ^24^, ^25^ However, we identified more males in all subgroups which is consistent with results from Zinzani et al.^11^ and Lucioni et al.,^24^ but contradicts findings from Hamilton et al.^8^

Lucioni et al. demonstrated that nodules and plaques are the two most common types of lesions in pcFCL and DLBCL‐LT, which is consistent with our findings.^24^

We noticed significant differences of elevated LDH levels between diseases groups (Figure 2) which were also found by Grange et al.^16^ To our knowledge, elevated LDH levels were more frequent in pcFCL than in pcMZLPD in our cohort which has not been reported so far in studies.

Hallermann et al. reported that three of 21 (14%) DLBCL‐LT patients had ulcerated nodules, whereas no ulcerations were found in pcFCL patients. In our cohort, 36% of patients with DLBCL‐LT showed ulcerations^26^.

Other cohorts showed comparable survival rates between 85–98%,^9^, ^10^, ^11^, ^16^, ^24^, ^25^, ^26^, ^27^ 90–100%^9^, ^10^, ^11^, ^25^, ^27^ and 14–73%^7^, ^8^, ^9^, ^10^, ^11^, ^18^, ^20^, ^24^, ^25^, ^26^, ^27^, ^28^ in pcFCL, pcMZLPD and DLBCL‐LT, respectively. Previous studies showed similar relapse rates between 14–47%^7^, ^8^, ^9^, ^10^, ^11^, ^16^, ^24^, ^25^ for pcFCL, 36–57%^7^, ^8^, ^9^, ^10^, ^11^, ^25^ for pcMZLPD and 16–85% ^7^, ^8^, ^9^, ^10^, ^11^, ^18^, ^20^, ^24^, ^25^ for DLBCL‐LT, respectively.

Based on TTNT, SDT appears to be more effective in pcFCL and pcMZLPD, whereas systemic treatments seem to be more beneficial in DLBCL‐LT. An exception is rituximab in pcFCL, with a TTNT of 50 months. However, rituximab was only administered to seven patients. A further sub‐analysis showed that two of three pcFCL patients with T3 classification received rituximab, while five of 28 pcFCL patients with T1 or T2 classification received rituximab. This is striking, because pcFCL patients with extended skin involvement were particularly selected for rituximab. Systemic rituximab has been also proposed as an effective and well‐tolerated treatment option for indolent CBCL entities,^13^, ^14^ which is consistent with the effectiveness of rituximab for our pcFCL patients with extended skin involvement. However, the mean TTNT was only 7 months of rituximab for our pcMZLPD patients.

Similarly, in mogamulizumab, an antibody‐dependent cellular cytotoxicity drug in CTCL, higher response rates were observed in patients with higher tumor burden.^29^ In univariate analysis, we observed that the lowest relapse rates were found in patients who underwent and received excision and rituximab, respectively. However, we cannot infer their efficacies due to the retrospective study design. Furthermore, treatment selection depends on patient‐centered features, physicians’ choices, hospitals’ standards and varying national guidelines.

In multivariate analysis, we found comorbidities to be favorable prognostic factors. Patients with comorbidities might be more compliant to treatment schemes which might result in a higher TTNT. Rice et al. investigated the impact of comorbidities on TTNT and survival in multiple myeloma. They reported that patients with asthma or chronic obstructive pulmonary disease (COPD) had a significantly longer median TTNT of the first line treatment compared to patients with neither asthma nor COPD.^30^ In multivariate analysis, leg involvement and pcFCL are unfavorable factors concerning the TTNT of the first line treatment. This analysis included all CBCL patients who presented with leg involvement, regardless of their disease entity. Since DLBCL‐LT patients present usually with leg involvement,^7^, ^8^, ^9^, ^10^, ^11^ this result may underscore the disease's aggressiveness. However, patients with pcFCL and pcMZLPD were also included in this analysis, which may also point out a negative impact of leg involvement in these patients.

Previous studies identified leg involvement as a negative prognostic factor, as well.^9^, ^16^, ^17^, ^18^ We found topical steroids as first line treatment, lower body involvement at diagnosis and secondary malignancies to be favorable prognostic factors affecting the number of relapses. The finding regarding topical steroids somewhat conflicts with the results shown in Table 2 and Figure 1, which depict the TTNT and RFS for topical steroids. However, this multivariate analysis assessed another endpoint than TTNT and RFS. Our finding about lower body involvement as a favorable factor somewhat contradicts studies which identified leg involvement as an unfavorable factor in multivariate analyses about patient survival.^9^, ^16^, ^17^, ^18^ Trunk involvement is an unfavorable prognostic factor in our cohort, which is consistent with findings from Smith et al.^17^ However, we assessed the endpoint number of relapses in our analysis.

In our cohort, the CLIPI did not show significant differences in RFS as opposed by Mian et al.^19^ The authors found the CLIPI to significantly impact the 5‐year progression‐free survival in the indolent entities of CBCL, allowing risk stratification among pcFCL and pcMZLPD patients.^19^ However, missing LDH values of 51 patients limited this analysis, as patients could have been underestimated regarding their CLIPI due to our adjustments made. Patients with potentially elevated LDH levels who have not undergone an assessment of LDH might have received a lower CLIPI leading to a bias in RFS.

Individualized treatment choices, even if made in multidisciplinary tumor boards, and the retrospective study design may limit the direct comparability between therapies. However, we believe the cohort's size and the inclusion of all treatment lines may strengthen the results, considering the disease's rarity.^21^ All patients were diagnosed based on clinicopathological correlation, which represents the current diagnostic gold standard. To our knowledge, TTNT has not been yet investigated in cohort studies about CBCL, along with triamcinolone. A case series reported on intralesional triamcinolone in CBCL suggesting its feasibility.^31^ This case series evaluated intralesional corticosteroids in nine CBCL patients, four of whom achieved complete remission. In the five remainder patients, partial responses were found.^31^

## CONCLUSIONS

In this study, we gained recent epidemiological insight into CBCL, assessing patient survival and identifying potential prognostic factors. This study is the first to examine treatment options and their efficacies in a large cohort of CBCL patients with the surrogate marker TTNT. Determining this emerging clinical endpoint may be a useful way of evaluating treatment options from a practical perspective in clinical setting.

Compared to systemic therapies, skin‐directed treatment showed superiority in pcFCL and pcMZLPD based on TTNT, whereas systemic treatments achieved higher TTNT in DLBCL‐LT. An exception is rituximab which had the longest TTNT in pcFCL patients with high tumor burden.

Currently, intralesional triamcinolone is not listed in the German S2k guidelines for cutaneous lymphoma.^32^ Triamcinolone is an established treatment option in dermatology which patients with hypertrophic scars, keloids, discoid lupus or cicatricial alopecia are commonly treated with. Due to its familiarity among dermatologists, triamcinolone seems to be an effective and well‐tolerated treatment in CBCL patients, as well. Therefore, this study delivers evidence for intralesional triamcinolone injections, which makes it a feasible treatment option, particularly for CBCL patients with multiple lesions.

Based on our results presented here, we endorse the follow‐up recommendations of the current German S2k guidelines for CBCL patients to assess possible changes in skin involvement.^32^ In pcFCL and pcMZLPD patients presenting with fewer than ten lesions, we recommend skin‐directed therapies, such as radiotherapy, excision or intralesional triamcinolone. We propose rituximab or chemotherapy, e.g. CHOP, possibly in combination with rituximab, in pcFCL and pcMZLPD patients with particularly high tumor burden, such as ten or more lesions, ulcerations or refractory patients to previous treatment lines. For patients with severely ulcerated nodules or DLBCL‐LT patients, we suggest CHOP or the chemotherapy consisting of cyclophosphamide, vincristine sulfate and prednisone (CVP) in elderly patients, which both can be combined with rituximab. Individualized decisions regarding radiotherapy for DLBCL‐LT patients may also be considered.

Due to the retrospective study design and occasionally missing data, we cannot draw definite conclusions about treatment options’ efficacy. However, the presented results might support clinical decision‐making.

Multicentric prospective studies are necessary to potentially overcome limitations of monocentric studies and allow generalizability, such as the *PROCLIPI* registry for CTCL^33^ or the *ADOReg* for skin cancers.^34^

## CONFLICT OF INTEREST STATEMENT

None.

## Supporting information



Supplementary information
