# Supplementary material for: How to treat primary cutaneous B cell lymphoma – Results from a monocentric cohort study on 98 patients
Source: J Dtsch Dermatol Ges. 2025 May 30;23(7):822–30. doi: 10.1111/ddg.15702 (PMC12257069; doi:10.1111/ddg.15702)
Supplement: Supplementary file 1 — Supplementary information [file DDG-23-822-s001.docx]

**Supporting information**

**Figure S1.** Kaplan Meier curve about leg involvement in primary cutaneous B cell lymphomas (univariate analysis)

**Figure S2.** Kaplan Meier curve about arm involvement in primary cutaneous B cell lymphomas (univariate analysis)

**Figure S1.** Kaplan Meier curve about leg involvement in primary cutaneous B cell lymphomas (univariate analysis)


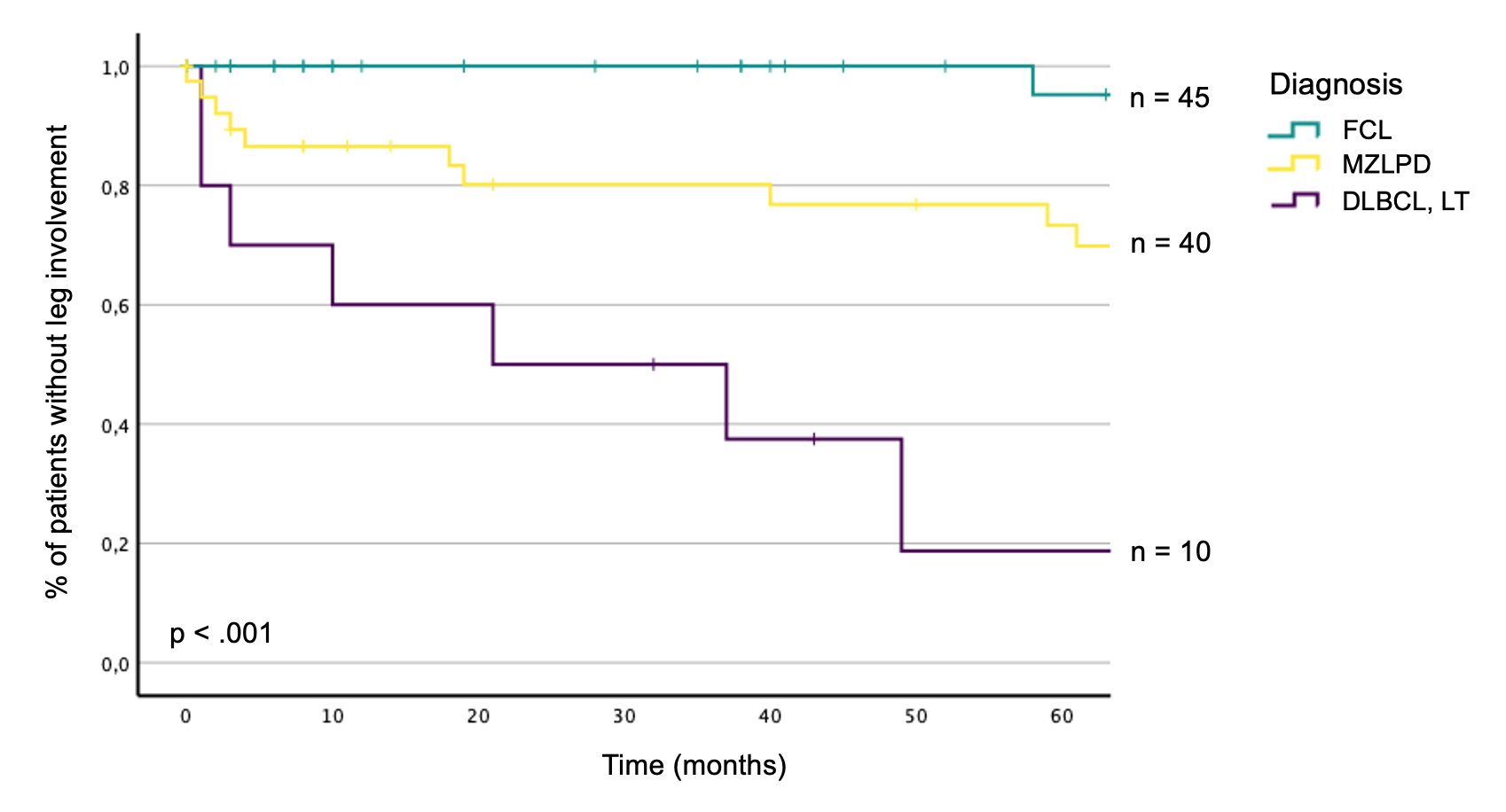


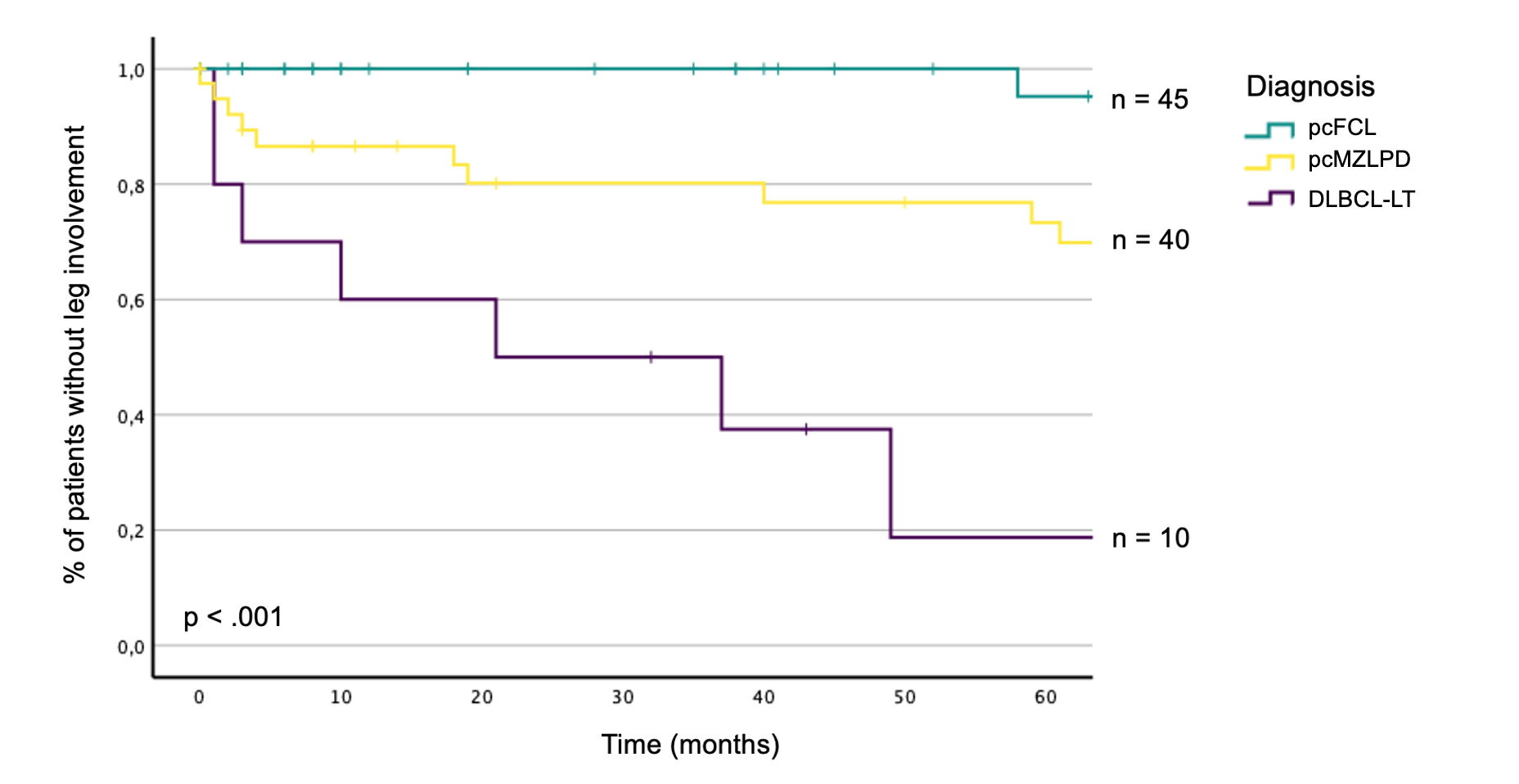
***Abbreviations:***

pcFCL, Primary cutaneous follicle center lymphoma.

pcMZLPD, Primary cutaneous marginal zone lymphoproliferative disorder.

DLBCL-LT, Diffuse large B-cell lymphoma, leg type.

p, P-value (Level of significance: α = 0.05).

n, Number of patients.

**Figure S2.** Kaplan Meier curve about arm involvement in primary cutaneous B cell lymphomas (univariate analysis)


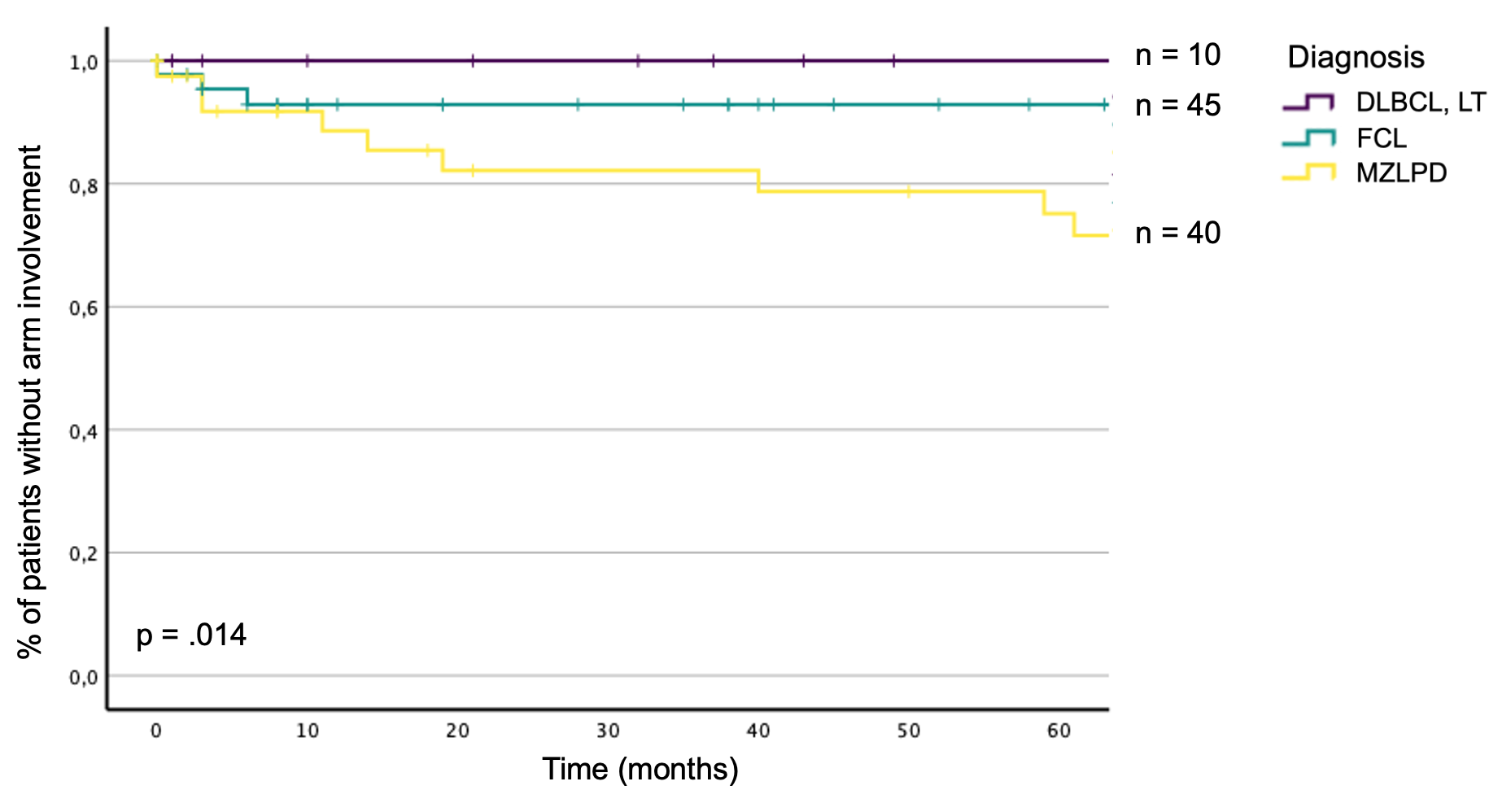


***
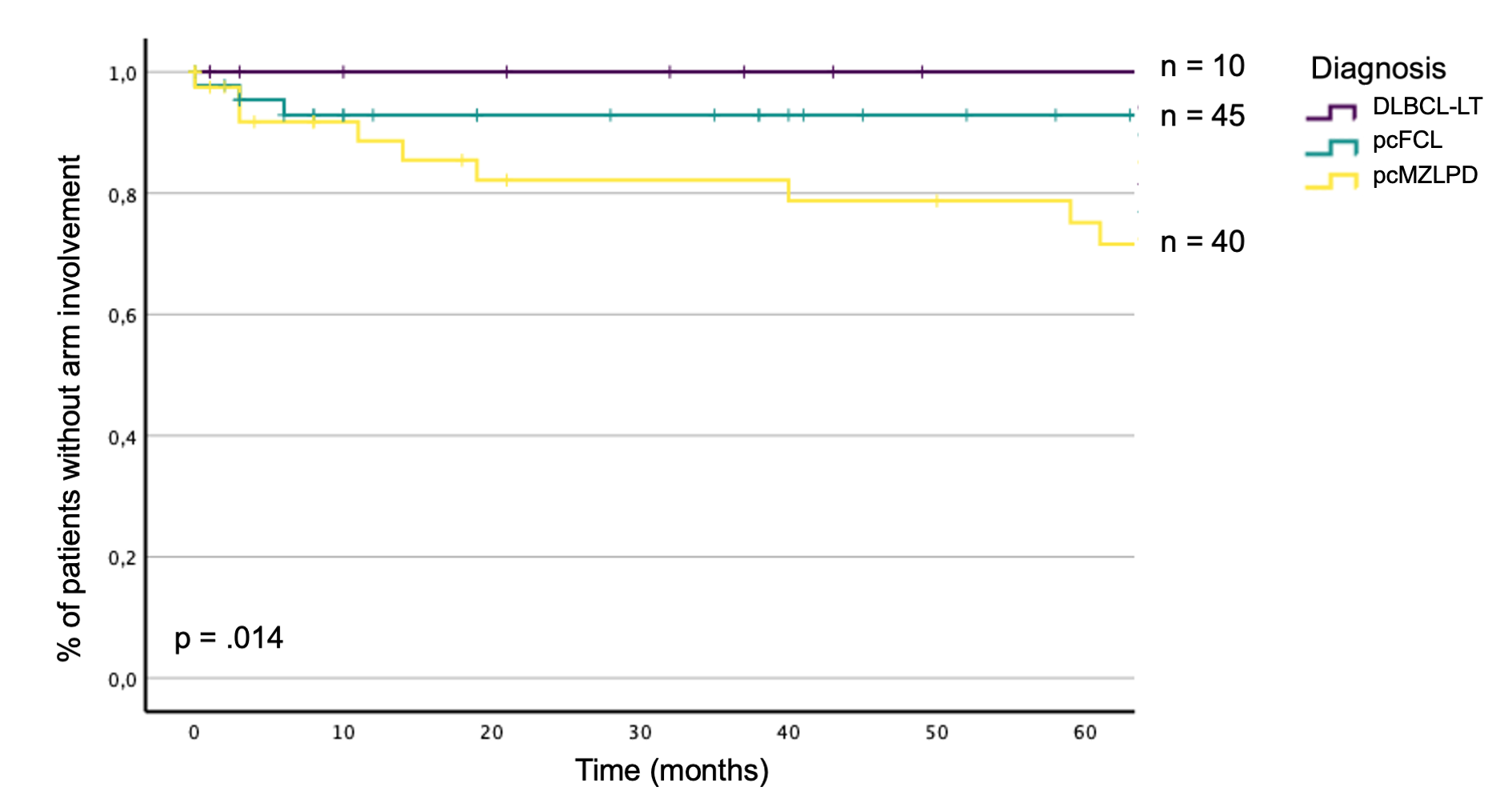
***

**Abbreviations:**

pcFCL, Primary cutaneous follicle center lymphoma.

pcMZLPD, Primary cutaneous marginal zone lymphoproliferative disorder.

DLBCL-LT, Diffuse large B-cell lymphoma, leg type.

p, P-value (Level of significance: α = 0.05).

n, Number of patients.
